# Supplementary material for: The Piezo channel is a mechano-sensitive complex component in the mammalian inner ear hair cell
Source: Nat Commun. 2024 Jan 16;15:526. doi: 10.1038/s41467-023-44230-x (PMC10791687; doi:10.1038/s41467-023-44230-x)
Supplement: Supplementary file 1 — Supplementary Information [file 41467_2023_44230_MOESM1_ESM.docx]

**The Piezo channel is a mechano-sensitive complex component in the mammalian inner ear hair cell.**

Jeong Han Lee*^1^, Maria C. Perez-Flores*^1^, Seojin Park^1,2^, Hyo Jeong Kim^1^, Yingying Chen^1^, Mincheol Kang^1,2^, Jennifer Kersigo^3^, Jinsil Choi^1^, Phung N. Thai^4^, Ryan L. Woltz^4^, Dolores Columba Perez-Flores^1^, Guy Perkins^5^, Choong-Ryoul Sihn^1^, Pauline Trinh^4^, Xiao-Dong Zhang^4^, Padmini Sirish^4^, Yao Dong^6^, Wayne Wei Feng^6^, Isaac N. Pessah^6^, Rose E. Dixon^7^, Bernd Sokolowski^8^, Bernd Fritzsch^3^, Nipavan Chiamvimonvat^4^, Ebenezer N. Yamoah^1#^

**Supplement Figures**

^1^Department of Physiology and Cell Biology, School of Medicine, University of Nevada, Reno, NV 89557 USA

^2^Prestige Biopharma, 11-12F, 44, Myongjigukje7-ro, Gangseo-gu, Busan, South Korea 67264

^3^Department of Biology, University of Iowa, Iowa City, IA, USA

^4^Division of Cardiovascular Medicine, Department of Internal Medicine, University of California, Davis, CA 95616, USA

^5^National Center for Microscopy and Imaging Research, University of California San Diego, La Jolla, CA 92093, USA

^6^ Department of Molecular Biosciences, School of Veterinary Medicine, University of California, Davis, 1089 VM3B, Davis, CA 95616, USA

^7^Department of Physiology & Membrane Biology, Tupper Hall, One Shields Avenue, Davis, CA. 95616, USA

^8^Department of Otolaryngology-Head and Neck Surgery, Morsani College of Medicine, University of South Florida, Tampa, FL, USA

Number of figures: 7

Supplement figures: 23

Number of pages: 90

Abstract: 146

Text:

#Corresponding author with complete address, including an email address:

*Authors contributed equally and are co-first authors.

Ebenezer N. Yamoah

Department of Physiology and Cell Biology

School of Medicine, University of Nevada Reno

1664 N Virginia St

Reno, NV 89557

enyamoah@gmail.com

**Supplement Figures**

**Supplement Figure 1**





**Supplement Figure 1 (S1). Negative and positive controls in cochlear sections.** smFISH localizes transcripts encoding the *Pz1/2* channels and *Tmc1* (**Fig. 1**), but not in negative control sections. **a-b**, Negative and positive probes provided by the manufacturer (ACD) were used on cryo-sections of the 2-week-old cochlea. Positive probes for mammalian samples were detected as fluorescent puncta in green and red. Outlines in red (left column) show one row of IHCs and three rows of OHCs. Comparison of control and experimental values are summarized in Fig. 1 and provided in the Results. Scale bar=10 μm.

**Supplement Figure 2**

**

**

**Supplement Figure 2 (S2).** **Salt bridge specificity is highly conserved in the Pz1 and Pz2 homotrimer interfaces**. **a-d**, Homotrimers of Pz1 (PDB: 6BPZ) from the extracellular (**a**) and transmembrane (**b**) side and Pz2 (PDB: 6KG7) from the extracellular (**c**) and transmembrane (**d**) side. Monomers are colored consistently in panels (**a-d**) with monomer 1=yellow, monomer 2=cyan, monomer 3=pink **e-f**) side view of a heterotrimer with a 2:1 (**e**) and a 1:2 (**f**) Pz1: Pz2 ratio. The protein backbone is shown in a ribbon with basic and acidic amino acids in the sphere. Coloring for Pz1 is as follows: ribbon=yellow, conserved basic=light blue, conserved acidic=red/orange, non-conserved basic or acidic=same color as protein ribbon. Pz2 coloring is as follows: ribbon=purple/pink, conserved basic=dark blue, conserved acidic=dark red, non-conserved basic or acidic=same color as protein ribbon.

**Supplement Figure 3**

**
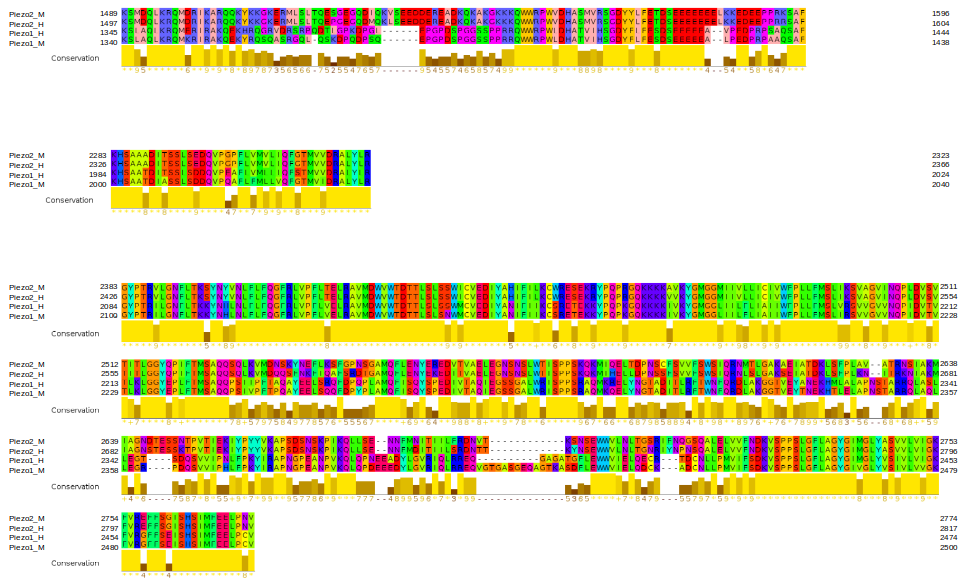
**

**Supplement Figure 3 (S3). Alignments of Pz1 and Pz2 interacting interfaces**.

Sequence alignment for Pz1 trimer interface. Alignment includes human Pz1 (Piezo1_H), mouse Pz1 (Piezo1_M), human Pz2 (Piezo2_H), mouse Pz2 (Piezo2_M). The sequence coloring is based on amino acid properties according to the "Taylor" scheme found in Jalview.

**Supplement Figure 4**

**

**

**Supplement Figure 4 (S4). Localization of Pz1 and Pz2 within nanometer proximity in hair cells**. **a-b.** The expression and distribution of Pz1 and Pz2 were detected using *Pz1-tdT* and *Pz2-GFP* (*Pz1-tdT/Pz2-GFP)* mice (P10) and assayed using a proximity ligation strategy. Images are shown as Z-projections through a stack of confocal micrographs Pz1 and Pz2 frequently co-localized within less than 40 nm proximity at the stereocilia and cuticular plate membrane in hair cells. The right panels are photomicrographs of the merged images. Scale bar=3 μm.

**Supplement Figure 5**

**

**

**Supplement Figure 5 (S5). Localization of Tmc1 and Pz2 in the IHC and OHC stereocilia.** **a,** Confocal fluorescence images obtained from *Tmc1-mCherry*:*Pz2-GFP* mice. Tmc1 (red) and Pz2 (green) labeling was strongest at stereocilia tips counter-stained in blue with Alexa-405-phalloidin for actin. Whole-mount cochlea from P10 *Tmc1-mCherry/Pz2-GFP* mice OHCs. The two rightmost panels illustrate Pz2 and Tmc1 labeling at the second (red arrow) and third (blue arrow) rows of OHC stereocilia. Labeling on the first row was rare. Scale bar=2 μm. **b,** Series of confocal sections at levels 0, 0.6, 1.2, and 1.8 μm, respectively, from stereocilia tips towards the cuticular plate at levels indicated. The inset in the left panel illustrates OHC and the sections' approximate levels marked with dashed red lines. Scale bar=3 μm. The rightmost panel is a side view of the OHC stereocilium showing the location of Pz2 and Tmc1 labeled with GFP and mCherry. Scale bar=0.5 μm. **c,** IHC stereocilia show expression of Pz2 (green) and Tmc1 (red). Scale bar=5 μm. **d,** Side view of IHC stereocilia showing Pz2 and Tmc1 location. Scale bar=0.7 μm. Scale bar for the rightmost panel=1 μm.

**Supplement Figure 6**

**

**

**Supplement Figure 6 (S6). Plasma membrane and cytoplasmic expression of mPz1 and mTmc1 in N2A cells. a.** A typical epifluorescent photomicrograph of an N2A cell 48 hrs post-transfection with *mPz1-Clover3* plasmid (left panel) and the corresponding image obtained upon switching to TIRF mode to determine membrane expression (right panel). **b.** Similar images were captured using *mTmc1-mRuby3* plasmid transfection. Scale bar (**a & b**)=10 μm. **c.** Shown is a summary plot of the ratio of the arbitrary fluorescence in TIRF mode (membrane localization) versus epifluorescence mode (cytoplasmic expression). Data were obtained from 15 cells. Comparing data from **a** and **b,** *p=0.039* (*n=15*).

**Supplement Figure 7**

**

**

**Supplement Figure 7 (S7). Mechanically activated (MA) Pz1 current (I_Pz1_) and pore-size estimation. a,** Representative traces of MA currents after *mPz1* expression in N2A cells, elicited by a series of mechanical steps of ~70-nm intervals at -80 mV holding voltage. **b,** Normalized current (I/I_max_)-displacement relationship of current fitted with a two-state Boltzmann function. **c,** Mean I-V relationship, using an instantaneous current-displacement protocol. The inset shows MA current traces evoked at different potentials (~1-μm displacement). Membrane potentials were stepped in 30-mV increments from -120 to +90 mV. **d,** Current-voltage plots normalized to the -90 mV current level for different internal amine compounds (left). Current obtained at 120 mV normalized to that at -120 mV plotted against the radius of the intracellular amine as determined from Corey-Pauling-Koltun (CPK) space-filling models. **e,** A plot of normalized current obtained at 120 mV with extracellular Na^+^ and various charge carriers against membrane potential (left). (**f, g**) The solid line represents estimates of inner and outer pore size with data fits showing a pore radius of 7±4 Å and 20±5 Å, respectively.

**Supplement Figure 8**

**

**

**Supplement Figure 8 (S8). Internal Ca^2+^ buffering altered Pz1-current (I_pz1_) decay kinetics but not mechanical sensitivity. a-b**, The current response traces from N2A cells transfected with *Pz1* to mechanical stimuli with different Ca^2+^ chelators, 5-mM EGTA, and BAPTA in the pipette solution. In black are the traces obtained with a hyperpolarized (-80 mV) holding potential, and in blue are traces evoked when cells were held at a depolarized (70 mV) holding potential. **c**, Summary box plot shows current decay (time constant (τ_decay_)) with different Ca^2+^ buffers. The τ_decay_ for maximum current (I_max_), elicited at a -80-mV holding potential in 5 mM EGTA (in ms), 24±3 (n=15), whereas in 5 mM BAPTA, it is 38±4 (n=5). The τ_decay_ at a 70-mV holding voltage in 10 mM EGTA is 71±8 (n=8), and in 10 mM BAPTA, it is 97±10 (n=6). There were significant differences (*p*<0.05) when comparing 5 mM EGTA (5-EGTA), BAPTA (5-BAPTA) at -80 mV, and 10 mM EGTA (10-EGTA) and BAPTA (10-BAPTA) at 70 mV, F(3,30)=(35), *p*=5.7X10^-10^. *Posthoc* comparisons using a Tukey HSD test indicate significant differences at -80 mV when comparing 5-EGTA vs. 5-BAPTA (*p*=1.3X10^-6^, and 10-EGTA vs. 10-BAPTA (*p*=0.02 at 70-mV when comparing 5-EGTA vs. 5-BAPTA (*p*=0.007) and 10-EGTA vs. 10-BAPTA (*p*=8.0X10-^6^) are significantly different. **d-e,** Normalized current-displacement plots at -80mV (black) and 70mV (blue), respectively. Internal solutions containing 5 mM EGTA are represented in squares, and 10 mM BAPTA in diamonds. Using a two-state Boltzmann equation to fit the data, half-maximum displacements X_1/2_ was ~0.85 μm, and the slope at X_1/2_ was ~50 pA μm^-1^. There were no significant differences between the two conditions.

**Supplement Figure 9**





**Supplement Figure 9 (S9). Alterations in mouse Piezo (mPz)-current properties after co-expression of Pz1, Pz2, Tmc1 (T1), and Tmc2 (T2). a**, Family of Pz1, Pz2, and co-transfected Pz1/2 (denoted; I_Pz1_, I_Pz2,_ and I_Pz1/2_) current traces invoked from N2A cell (total DNA, 1 μg). For the *Pz1/2,* a 1:1 molar ratio was used (total, 1 μg). Cells were held at -80 mV. Despite sustained mechanical displacement, visible differences were apparent in the rapid activation and decay. Co-expressed *Pz1/Pz2* plasmid yielded a hybrid current. **b,** Summary of the displacement-response of I_Pz1_ (■), I_Pz2_ (●), and I_Pz1/2_ (▲). Uniformed sigmoidal curves generated from a two-state Boltzmann function showed that the half-activation (X_1/2_, (in μm)) was 0.87±0.01 (n=11), I_Pz2_ was 0.67±0.01 (n=10) and I_Pz1/2_ was 0.76±0.01 (n=12). Displacement, (X_1/2_, pA μm^-1^) sensitivity for I_Pz1_ was 80±8 (n=11), I_Pz2_ was 54±15 (n=10), and I_Pz1/2_ was 72±6 (n=12). **c,** Representative maximum current traces recorded from N2A cells 48-hrs after transfection with *Pz1, Pz2, Pz1/2, Pz1/Tmc1 (T1),* and *Pz1/Tmc2 (T2)* plasmids. The respective decay time constants (τ) are noted for each trace. **d,** Summary τ_decay_ data for the maximum current for the five transfection conditions. Mean τ_decay_ (in ms) I_Pz1_ = 25±6 (n=14), I_Pz2_ = 6±2 (n=13), I_Pz1/2_ = 17±5 (n=14), I_Pz1/T1_ = 13±3 (n=14) and I_P1/T2_ = 6±3 (n=14). There were significant differences at *p*<0.05 level for the τ_decay_ of the different transfection conditions F(4,64)=(53) *p*=1.4X10^-19^. *Post hoc* comparisons τ_decay_ using the Tukey HSD test indicate that I_Pz1_ vs. I_Pz1/2_ (*p*=1.6X10^-4^); I_Pz2_ vs. I_Pz1/2_ (*p*=3.9X10^-8^); I_Pz1_ vs. I_Pz1T1_ (*p*=3.6X10^-8^); I_Pz1_ vs. I_Pz1T2_ (*p*=1.1X10^-30^); and I_Pz1T1_ vs I_Pz1T2_ (*p*=1.2X10^-4^) are significantly different. **e,** Co-transfection of *Pz1* and *Tmc1 (T1)* plasmid resulted in a profound increase in the MET current compared with *Pz1* and *Tmc2 (T2)* or *Pz1* alone in N2A cells. Typical I_Pz1_ and I_Pz1/T1_ traces are shown as an inset. The maximum current Imax is 40-42 hrs post-transfection for Pz1, Pz1/T1, and Pz1/T2. Mean values (in pA) for I_Pz1_=1127±488 (n=15), I_Pz1/T1_ = 2920±1069 (n=15), I_Pz1/T2_ = 1049±569 (n=15). There were significant differences at *p*<0.05 level for the I_max_ of the different transfection conditions F(2,42)=(30) *p*=9.8X10^-9^. *Post hoc* comparisons I_max_ using the Tukey HSD test indicate that I_Pz1_ vs. I_Pz1T1_ (*p*=1.6X10^-7^); I_Pz1/T1_ vs. I_Pz1/T2_ (*p*=2.8X10^-8^) are significantly different, but I_Pz1_ vs. I_Pz1/T2_ (p=1.0) is not significantly different. **f,** Summary of the displacement-response of I_Pz1_ (■), I_Pz1/T1_ (●), and I_Pz1/T2_ (●). Uniformed sigmoidal curves generated from a two-state Boltzmann function. For I_Pz1_, the half-activation (X_1/2_, (in μm)) = 0.83±0.02 (n=11), I_Pz1/T1_ = 0.88±0.01 (n=10) and I_Pz1/2_ = 0.86±0.01 (n=12).

**Supplement Figure 10**

**
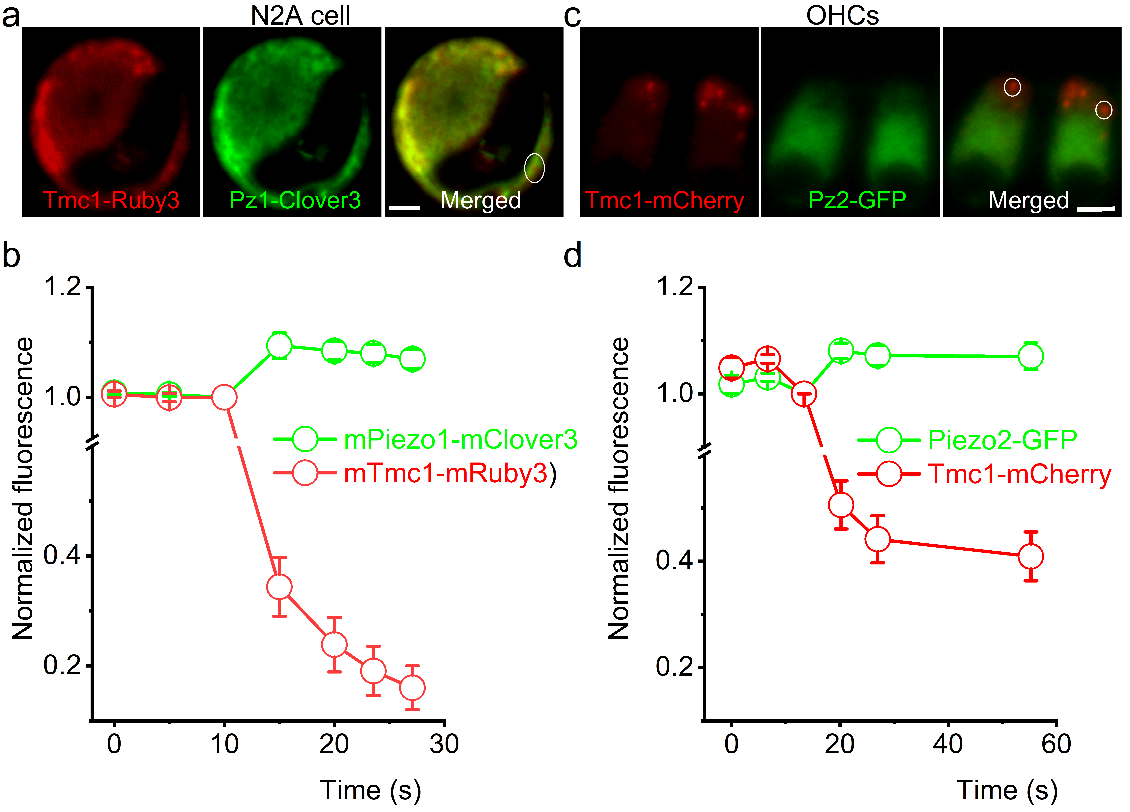
**

**Supplement Figure 10 (S10)**

**Interactions between mPz1 and mTmc1 in N2A cells and Pz2 and Tmc1 in OHCs.** mPz1-mClover3 (donor) and mTmc1-mRuby3 (acceptor), co-transfected and co-expressed in N2A cells. The time courses showed the donor fluorescent intensity signal enhancement when the fluorescent acceptor signal was photobleached, showing the functional interaction and proximity of the mPz1 and mTmc1. **a & c.** Photomicrographs of N2A cells expressing mPz1-mClover3 and mTmc1-mRuby3. (**a**) and mouse OHCs expressing Pz2-GFP and Tmc1-mCherry from a mouse. The white circle indicated a region of interest (ROI) where recordings were made. OHCs were isolated from Piezo2-GFP (donor) and Tmc1-mCherry (acceptor) transgenic mice. **b & d.** The time course showed the enhancement of the donor fluorescent intensity signal when the fluorescent acceptor signal was photobleached, demonstrating the interaction and proximity of the mPz1 and mTmc1 in N2A cells and mPz2 and mTmc1 in OHCs. The mean FRET efficiency (E) in N2A cells was 0.10±0.02 (n=8). In OHCs, E was 0.10±0.01 (n=7). **a & c,** Scale bar = 5 μm.

**Supplement Figure 11 (S11)**

**Supplement Figure 11 (S11)**

**Dihydrostreptomycin (DHS) block of I_Pz_ and I_Pz/Tmc1_**

**a,** Mechanically-activated (MA) current in N2A cells expressing Pz1 and evoked at -80 mV with 1-μm displacement in control (black) after 100-μM (gray) and 1000-μM (light gray) DHS application. **b,** MA current traces under control and after the indicated DHS application. Cells were held at -80 mV and activated using 1-μm displacement. Traces in black, green, purple, blue, and cyan were control (in μM) 0.05, 0.1, 1, and 2, respectively. **c,** DHS concentration-inhibition response for I_Pz1_ IC_50_=270±135 μM (n=7), and I_Pz1/Tmc1_=70±6 nM (n=7). Data from neomycin blocked (200 μM) indicated (∆) ~65% of the total I_Pz1_ (n=2).

**Supplement Figure 12**

**

**

**Supplement Figure 12 (S12)**

**Pz1 single-channel activity.**

**a,** Family of unitary single-channel traces recorded from N2A cell patch under at -80 mV holding potential subjected to -4-mm Hg pressure. **b,** The amplitude histogram of the relative counts versus unitary current amplitude. **c,** Unitary current amplitude determined at different holding potentials assessed from 5 patches from 5 N2A cells and fitted with a linear regression line. The single-channel conductance (γ) was 36±5 pS (n=5).

**Supplement Figure 13**

**

**

**Channel properties of purified Piezo reconstituted in a planar lipid bilayer.** Induced incorporation of Pz1 channels into the bilayer lipid membrane (BLM) from the cis side was recorded at a potential of 100 mV (cis to trans). The recording buffer was symmetric (500-mM KCl, both cis and trans). Channel activities were recorded at the discrete holding potentials indicated or during a programmed voltage ramp protocol. **a-d**, Representative current traces of recordings made from independent channel incorporations at the indicated holding potentials using BLMs formed with synthetic lipid (PE:PS: PC 5:3:2; w/w); The dashed line(s) and their corresponding numbers to the right of the traces indicate the number of channels resolved. The unitary conductance (γ or G_o_) was obtained from a linear fit from the current/voltage (I/V) relationships (**e**). The results presented in **a-f** were from 2 separate protein preparations. Channel activities recorded with BLM formed with brain total lipid extract are shown in **f-k**. The representative current traces of a 4-channel recording under the indicated holding potentials are shown in **f.** The dashed oval indicates the region of the trace expanded in the inset immediately above (recordings made at 40 mV). The unitary conductance (γ or G_o_) is shown in **g**. Channel activities were recorded before (control period) and following the addition of (**h**) 100 μM DHS, (**i**) 1 mM or 2 μM GsMTx4, or (**j**) 20 μM ruthenium red, non-selective ion channel blockers under a voltage ramp protocol (**k**). The results presented in **f-k** were from 2 separate protein preparations.

**Supplement Figure 14**

**

**

**Supplement Figure 14 (S14). The mechanically-activated (MA) current is generated by wild-type and mutant Pz1 and Pz2 channels. a, (left panel)** Wild-type *Pz1* with the amino acid at the C-terminal domain (CTD) MFEE at 2493-2496 expressed in N2A cells. Typical MA current traces were generated with stepped displacements from 0 to 2 μm ΔX=0.4 μm. Panels indicated show current traces generated with similar protocols from N2A cells expressing AAAA amino acid mutation, wild-type *Pz1*, MFEE:AAAA at a ratio of 1:1, and *Pz2*, MFEE:AAAA (1:1). For *Pz2*; the MFEE was at position 2767-2770. **b,** Summary data comparing the maximum current recorded from N2A cells expressing wild-type and mutant *Pz1* and *Pz2*. Mean maximum current for wild-type (in pA) Pz1-MFEE=1156±142, n=15; Pz1-AAAA=14±4, n=15; Pz1-MFEE: AAAA-Pz1=79±9, n=15; Pz1-MFEE: AAAA-Pz2=63±9, n=15. One-way ANOVA, F(3,56)=(60) *p=1.5X10^-17^*; *Posthoc* comparisons Pz1-MFEE vs Pz1-AAAA *p=3.8X10^-7^;* Pz1-MFEE vs Pz1-MFEE:AAAA-Pz1 *p=9.5X10^-8^,* and Pz1-MFEE vs Pz1-MFEE:AAAA-Pz2 *p=9.6X10^-8^.*

**Supplement Figure 15**

**

**

**Supplement Figure 15 (S15): *Myo15-Cre (mc)* and *Calb2-Cre* (cc) genotype analyses and expression.**

**a-b,** PCR genotyping of wild-type and mutant littermate mice for *Myo15-Cre* and *Calb2-Cre* lines. **c,** tdT (red) expression in IHC and OHCs from *Myo15-Cre-Ai9-tdT* P5 mice shows cell-specific identification of the Cre-line. **d,** Postnatal calretinin (Calb2-GFP gene) in mice at P5 shows robust expression in IHCs and moderate expression in OHCs. Myo7a is used as a hair cell marker in cyan, and actin is labeled with phalloidin (red). Scale bar=10 μm.

**Supplement Figure 16**

**

**

**Supplement Figure 16 (S16). Body weight of wild-type (WT) and expression of Pz1 in *Pz-ki* mice. a,** Mean body weight of control (*myo15-cre (mc)* and *calb2-cre (cc)*) and mc-*Pz1^MU^, mc-Pz2^MU^* and cc-*Pz2^MU^* mice. Data are shown as a box plot with scattered intervals and listed as mean±SD. Equal numbers were tested for males and females. When odd numbers are reported, the females outnumber males. No significant differences were detected between the sexes. Thus, the reported data were combined. For *myo15-cre* and *Pz* *ki* lines one-way ANOVA, F(5,78)=(21) *p*=*2.0X10^-13^*. *Post hoc* comparisons using the Tukey HSD test indicate that at 4 weeks (body weight in grams (g)), *myo15-cre (mc)* (23±1, n=17) *vs.* mc-*Pz1^MU^* (14±1, n=19) (*p*=4.9X10^-8^); *mc* (23±1, n=18) *vs.* mc-*Pz2^MU^* (14±1, n=12) (*p*=1.2X10^-7^) are significantly different. For *Calb2-Cre (cc)* and the *Pz^MU^* lines, one-way ANOVA, F(3,40)=(3) *p*=0.03. *Post hoc* comparisons using the Tukey HSD test indicate that at 4-week *cc* (19±1, n=8) vs. cc-*Pz2^MU^* (21±1, n=11) (*p*=0.3) are not significantly different. **b**, Immunofluorescence labeling of OHCs of a P21 whole-mount cochlea showing positive reactivity of actin (green), Pz1 (red), and Myo7a (cyan) antibodies. The merged image is shown. Scale bar = 5 μm.

**Supplement Figure 17**

**

**

**Supplement Figure 17 (S17). Progression of ABR thresholds to tone pip sounds and clicks from 4- 8- week-old *mc-Pz1/2^MU^* and *mc-Pz1-2^MU^* mice.**

**a-c**, Summary of the ABR thresholds to tone pip sounds. **a,** Comparison of ABR thresholds for *mc, mc-Pz1^MU^* in 4- and 8-week-old mice. Mice were littermates, and male and female numbers were equal. When odd numbers are reported, the females outnumber males. We combined the data since no significant differences were detected between the sexes. Data are shown as mean ± SD. For 4 kHz, sound threshold (in dB) *mc* and mc-*Pz1^MU^* lines one-way ANOVA, F(2,33)=(21) *p*=*1.4X10^-6^*. *Post hoc* comparisons using the Tukey HSD test indicate that at 4-w (4 kHz), *mc* (51±9, n=16) vs. *mc-Pz1^MU^* (74±15, n=12) (*p*=*2.3X10^-5^*); at 8-w *m-c* (55±4, n=14) vs. *mc-Pz2^MU^* (78±9, n=8) (*p=1.5X10^-5^*) are significantly different. For 8 kHz, *mc* and *mc-Pz1^MU^* lines one-way ANOVA, F(2,28)=(20) *p=4.6X10^-6^.* *Post hoc* comparisons using the Tukey HSD test indicate that at 4-w (8 kHz), *mc* (43±13, n=12) *vs.* *mc-Pz1^MU^* (65±16, n=12) *p*=*5.0X10^-4^*; at 8-w *mc* (45±6, n=8) *vs.* mc-*Pz1^MU^* (79±7, n=7) *p=5.9X10^-6^* are significantly different. For 16 kHz, *mc* and mc-*Pz1^MU^* lines one-way ANOVA, F(2,27) = (56) *p*=*2.5X10^-10^*. *Post hoc* comparisons using the Tukey HSD test indicate that at 4-w (16 kHz), *mc* (31±8, n=10) *vs.* *Pz1-ki^m-c^* (59±10, n=12) (*p*=2.5X10^-10^); at 8-w *mc* (30±2, n=9) *vs.* mc-*Pz1^MU^* (76±10, n=8) (*p*=*1.2X10^-12^*) are significantly different. For 32 kHz, *mc* and mc-*Pz1^MU^* lines one-way ANOVA, F(2,34)=(9) *p=6.5X10^-4^*. *Post hoc* comparisons using the Tukey HSD test indicate that at 4-w (32 kHz), *mc* (71±9, n=16) *vs.* mc-*Pz1^MU^* (82±11, n=12) (*p*=*8X10^-3^*); at 8-w *mc* (76±2, n=7) *vs.* mc-*Pz1^MU^* (86±5, n=9) (*p=1.3X10^-3^*) are significantly different. **b,** Using similar assessment for *mc* and *mc-Pz2^MU^* 4 kHz, sound threshold (in dB) *mc* and *mc-Pz2^MU^* lines one-way ANOVA, F(2,43)=(39) *p*=*2.1X10^-10^*. *Post hoc* comparisons using the Tukey HSD test indicate that at 4-w (4 kHz), *mc* (56±11, n=17) *vs.* *mc-Pz2^MU^* (80±10, n=20) *p=1.0X10^-20^*; at 8-w *m-c* (55±6, n=16) *vs.* *mc-Pz2^MU^* (85±6, n=9) p=*1.1X10^-20^* are significantly different. For 8 kHz, *mc* and *mc-Pz2^MU^* lines one-way ANOVA, F(2,38)=(24) *p*=2.3X10^-7^. *Post hoc* comparisons using the Tukey HSD test indicate that at 4-w (8 kHz), *mc* (46±14, n=12) *vs.* *mc-Pz2^MU^* (77±15, n=20) (*p*=*6.8X10^-7^*); at 8-w *mc* (45±14, n=12) *vs.* *mc-Pz2^MU^* (79±9 n=9) (*p*=*5.9X10^-6^*) are significantly different. For 16 kHz, *mc,* and *mc-Pz2^MU^* lines one-way ANOVA, F(2,37)=(44) *p*=*1.6X10^-10^*. *Post hoc* comparisons using the Tukey HSD test indicate that at 4-w (16 kHz), *mc* (30±7 n=11) *vs.* *mc-Pz2^MU^* (68±17, n=20) (*p*=1X10-20); at 8-w *mc* (29±3, n=11) *vs.* *mc-Pz2^MU^* (86±5, n=9) *p=1.1X10^-20^* are significantly different. For 32 kHz, *mc* and *mc-Pz2^MU^* lines one-way ANOVA, F(2,41)=(16) *p*=*7.4X10^-6^*. *Post hoc* comparisons using the Tukey HSD test indicate that at 4-w (32 kHz), *mc* (69±9, n=17) *vs.* *mc-Pz2^MU^* (82±9, n=18) *p=8.9X10^-5^*; at 8-w *m-c* (72±2, n=13) *vs.* *mc-Pz2^MU^* (86±5, n=9) *p*=*5.4X10^-5^* are significantly different. **c**, ABR thresholds *mc* and double knockin mice, *mc-Pz1-2^MU^* at 4 kHz, sound threshold (in dB) *mc* and *mc-Pz1-2^MU^* lines one-way ANOVA, F(2,24) = (50) *p*=*2.9X10^-9^*. *Post hoc* comparisons using the Tukey HSD test indicate that at 4-w (4 kHz), *mc* (57±9, n=9) *vs.* *mc-Pz1-2^MU^* (83±7, n=9) *p=7.8X10^-8^*; at 8-w *mc* (56±10, n=9) *vs.* *mc-Pz1-2^MU^* (88±4, n=9) p=*1.0X10^-20^* are significantly different. For 8 kHz, *mc* and *mc-Pz1-2^MU^* lines one-way ANOVA, F(2,24)=(31) *p*=2.0X10^-7^. *Post hoc* comparisons using the Tukey HSD test indicate that at 4-w (8 kHz), *mc* (48±15, n=9) *vs.* *mc-Pz1-2^MU^* (78±8, n=9) (*p*=*4.9X10^-6^*); at 8-w *mc* (47±13, n=9) *vs.* *mc-Pz1-2^MU^* (83±6 n=9) (*p*=*3.7X10^-7^*) are significantly different. For 16 kHz, *mc* and *mc-Pz1-2^MU^* lines one-way ANOVA, F(2,24)=(270) *p*=*3.6X10^-17^*. *Post hoc* comparisons using the Tukey HSD test indicate that at 4-w (16 kHz), *mc* (27±7 n=9) *vs.* *mc-Pz1-2^MU^* (81±7, n=9) (*p=1X10^-20^*); at 8-w *mc* (29±3, n=9) *vs.* *mc-Pz1-2^MU^* (86±3, n=9) *p=1.1X10^-20^* are significantly different. For 32 kHz, *mc* and *mc-Pz1-2^MU^* lines one-way ANOVA, F(2,24)=(23) *p*=2*.3X10^-6^*. *Post hoc* comparisons using the Tukey HSD test indicate that at 4-w (32 kHz), *mc* (72±8, n=8) *vs.* *mc-Pz1-2^MU^* (86±5, n=9) *p=7.7X10^-5^*; at 8-w *mc* (74±2, n=9) *vs.* *mc-Pz1-2^MU^* (89±2, n=9) *p*=*3.1X10^-6^* are significantly different. For click, *mc* and *mc-Pz1-2^MU^* lines one-way ANOVA, F(2,24)=(132) *p*=*1.2X10^-13^*. *Post hoc* comparisons using the Tukey HSD test indicate that at 4-w (click), *mc* (46±7 n=9) *vs.* *mc-Pz1-2^MU^* (83±7, n=9) (*p=1X10^-20^*); at 8-w *mc* (49±5, n=9) *vs.* *mc-Pz1-2^MU^* (87±3, n=9) *p=1.1X10^-21^* are significantly different.

**Supplement Figure 18**

**

**

**Supplement Figure 18 (S18). Reduced FM1-43 uptake in *Pz^MU^* hair cells**

**a,** Fluorescent images of FM1-43 were taken at different time points at three focal levels, L1, L2, and L3, referring to the apical, cuticular plate, and basal levels, respectively. The frames shown are at L2 (cuticular plate). Time 0 indicates the onset of dye application (10 μM for the 5-sec duration). Consecutive images of the bundles at stereocilia, cuticular plate, and basal levels were taken in 5-second intervals. The dye enters the apical aspects of the cell before being visualized at the basal pole. (Scale bar=10 μm). **b,** The change in fluorescence at L2 (cuticular plate) focal levels as a function of time (adjusted for the interval between frame capture at each level). Densitometric data of mean pixel intensity were measured in arbitrary grayscale units (a.u) as described^19^. The number of animals tested is indicated for controls and genotypes. Frames were taken at the three rows of OHCs at the apical one-third of the cochlea, and a similar loading pattern is observed at the middle third of the cochlea. The change in fluorescence was fitted with an exponential function, and the time constants (τ, in secs) of FM1-43 dye loading in control, *mc-Pz1^MU^*, and *mc-Pz2^MU^* apical cochlear OHCs at L2, 23+3 (n=5), 61+6 (n=4) and 58+8 (n=4).

**Supplement Figure 19**

**

**

**Supplement Figure 19 (S19). Cellular degeneration of *Pz^MU^* mice. a,** Whole-mount cochlea of a ~6-week-old *Calb2-Cre (cc)* control mouse showing the low-frequency segment at the ~6-kHz cochlear region. Myo7a, the hair cell marker, is stained (white), and the stereocilia marker is stained (green) for actin. Scale bar = 10 μm. **b,** The high-frequency segment at the ~32-kHz cochlear region**.** ABR thresholds for the mouse were (dB): 4-kHz=40, 8-kHz=20 dB, 16-kHz=15, 32-kHz=60, and click=35. **c-d,** A 6-week-old cc-*Pz2^MU^* cochlea shows 6 and 32 kHz segments. Note enlarged IHCs (red arrows) at the low-frequency ~6-kHz segment and lost OHCs (* in red) at the high-frequency ~32-kHz segment. Recorded ABR thresholds for the mouse were (dB): 4-kHz=60, 8-kHz=45, 16-kHz=85, 32-kHz=90, and click=55. Scale bar=10 μm.

**Supplement Figure 20**

**

**

**Supplement Figure 20 (S20). Hair cell loss and degeneration in *Pz^MU^* mice as observed with SEM at P56. a,**The *mc-Pz2^MU^* cochlear apex reveals near-normal IHCs and degenerating OHCs. **b-c,** The degenerating OHCs have fused stereocilia or progressive loss. **d,** Profound IHC and OHC loss in the *Pz^MU^* cochlear base. **e-f,** There are only a few remaining IHCs. Scale bar = 10 µm and 2 µm (**b,c**).

**Supplement Figure 21**

**
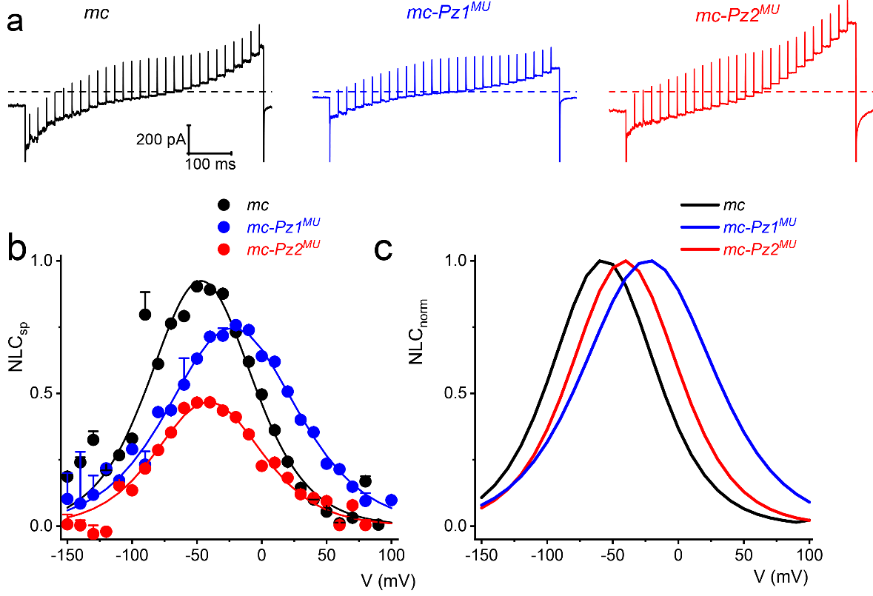
**

**Supplement Figure 21 (S21). Voltage-dependent nonlinear capacitance (NLC) in OHCs in *Myo15-Cre (mc)* and *mc-Pz1/2^MU^* mice.** **a**, Upper panels show representative traces of NLC recordings using a voltage stair protocol ranging from −150 to +100 mV with 10-mV increments. Left panel (control (black)), myo15-cre (m-c), middle panel (mc-*Pz1^MU^* (blue)), and right panel (*mc-Pz2^MU^)* (red)). **b,** Normalized NLC, plotted as a function of voltage and fitted with the first derivative of a Boltzmann function describing nonlinear charge movement^74^. For OHC cells from *mc*, *mc-Pz1^MU^,* and mc-*Pz2^MU^* mice (n=5 for each group). NLC obtained through correction for linear capacitance was plotted as a function of voltage and fitted with the first derivative of a Boltzmann function: C_m_ = C_ln_ + C_v_ = C_ln_ + (Q_max_ze/kT) × exp(−ze[V − V_h_]/kT)/(1 + exp[−ze(V − V_h_)/kT])^2^, where C_m_ is the total capacitance of the cell, C_ln_ is the linear capacitance, C_v_ is the nonlinear capacitance, V is the membrane potential, V_h_ is the voltage at half-maximal nonlinear charge transfer, e is the electron charge, k is Boltzmann's constant, T is the absolute temperature, z is the valence, and Q_max_ is maximum nonlinear charge transfer. For the plots shown, values for *myo15-cre* (*mc*; controls), (C_ln_ (pF), z, Q_sp_ (fC/pF) and V_h_ (mV) were (9, -0.9, 120, -47); *mc-Pz1^MU^,* (7, -0.8, 98, -23); and *mc-Pz2^MU^*, (10, -0.9, 50, -40). **c,** Normalized NLC illustrates the shifts in V_h,_ comparing controls with the *Pz^MU^* OHCs.

**Supplement Figure 22**

**
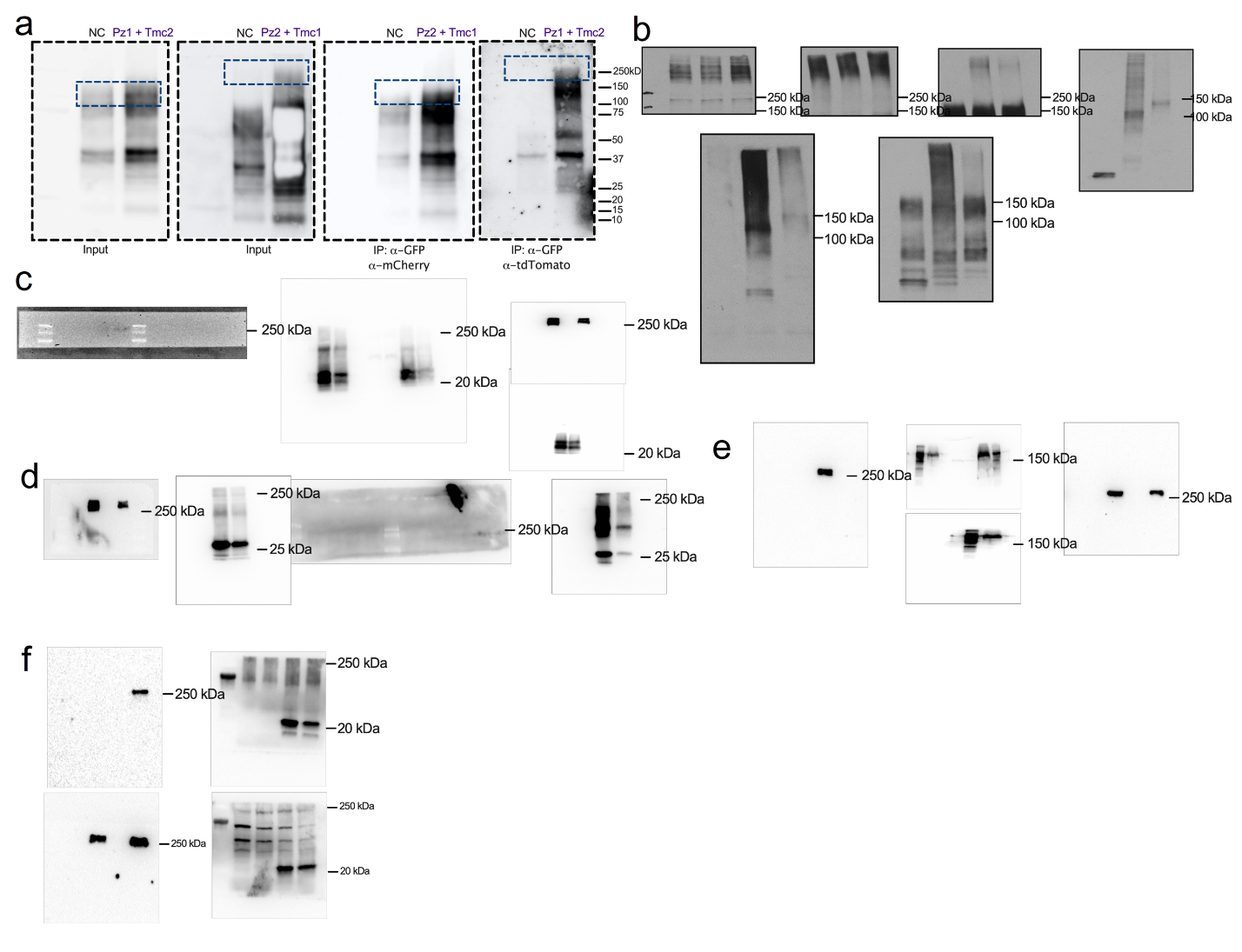
**

**Supplement Figure 22 (S22).**

**Original gels showing Pz1/2 exists in a complex with Tmc1/2 in cochlear tissue and forms a protein complex with MET complex proteins (see Fig. 7). a**. Immunoblots of GFP input and tdTomato and mCherry after IP. **b,** Co-immunoprecipitation of FLAG-tagged Pz1 and mCherry-tagged Tmcs. Immunoprecipitation and western blot analysis were performed with anti-flag and anti-mCherry antibodies of a cell transfected with various combinations as listed (Lane 1: mCherry + Pz1-FLAG, lane 2: Tmc1-mCherry + Pz1-FLAG, lane 3: Tmc2-mCherry + Pz1-FLAG). Lane 1 showed a negative control. (**c**), Lhfpl5-Myc, and Pz1-FLAG (**d**), Pcdh15-HA and Pz1-FLAG (**e**) Cib2-V5 and Pz1-FLAG (**f**) and Tmc1- mCherry and Pz1-FLAG (**e**). Proteins were extracted from HEK 293 cells that transiently expressed target proteins. (**f**), Pull-down samples were detected using α-FLAG to identify Pz and α-mCherry to detect Tmc1 (**b**) α-His to detect Tmie (**c**), α-Myc to detect Lhfpl5 (**d**), α-HA to detect Pcdh15 (**e**), α-V5 to detect Cib2 (**f**) in western blotting.

**Supplement Figure 23**

**
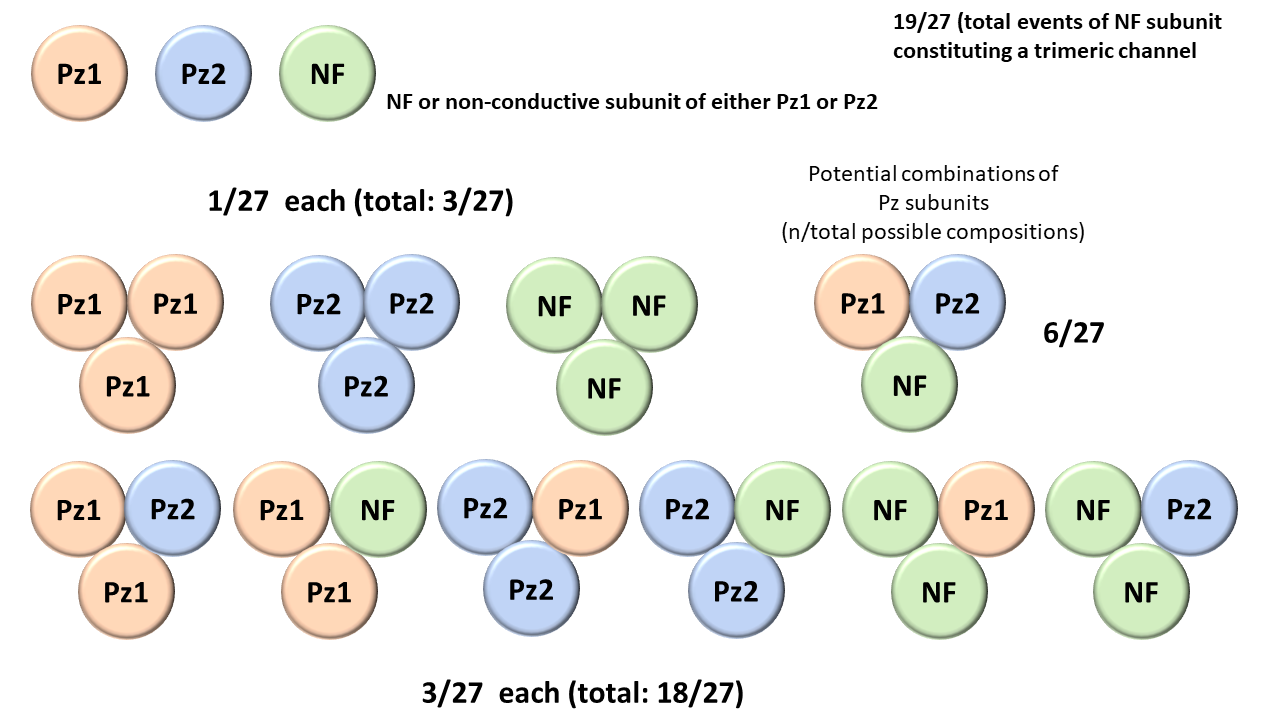
**

**Supplement Figure 23 (S23). Predicted multimerization of endogenous/wild-type Pz1, Pz2, and non-functional Pz mutant subunits.** Schematic of expected channel subunit combinations with wild-type (WT) Pz1 and AAAA mutant (MU) Pz subunits. The numbers at the bottom show the potential membrane-expressed channel populations formed from each ratio of WT and MU subunits. For a stochastic assembly of channel subunits, and assuming functional hetero- and homomeric channels and equal levels of subunit expression, ~30% of HCs may carry functional Pz subunits, i.e., ~70% carry mutant channels, whereas the number of functional HCs decreases to ~12% in the *Pz1-2^MU^* cochlear HCs.
