# Supplementary material for: The Piezo channel is a mechano-sensitive complex component in the mammalian inner ear hair cell
Source: Nat Commun. 2024 Jan 16;15:526. doi: 10.1038/s41467-023-44230-x (PMC10791687; doi:10.1038/s41467-023-44230-x)
Supplement: Supplementary file 3 — Reporting Summary [file 41467_2023_44230_MOESM3_ESM.pdf]

nature portfolio

Corresponding author(s): Ebenezer N. Yamoah

Last updated by author(s): Dec 1, 2023

Reporting Summary

Nature Portfolio wishes to improve the reproducibility of the work that we publish. This form provides structure for consistency and transparency in reporting. For further information on Nature Portfolio policies, see our Editorial Policies and the Editorial Policy Checklist.

Statistics

For all statistical analyses, confirm that the following items are present in the figure legend, table legend, main text, or Methods section.

n/a	Confirmed	
☐	☒	The exact sample size (*n*) for each experimental group/condition, given as a discrete number and unit of measurement
☐	☒	A statement on whether measurements were taken from distinct samples or whether the same sample was measured repeatedly
☐	☒	The statistical test(s) used AND whether they are one- or two-sided<br>*Only common tests should be described solely by name; describe more complex techniques in the Methods section.*
☐	☒	A description of all covariates tested
☐	☒	A description of any assumptions or corrections, such as tests of normality and adjustment for multiple comparisons
☐	☒	A full description of the statistical parameters including central tendency (e.g. means) or other basic estimates (e.g. regression coefficient) AND variation (e.g. standard deviation) or associated estimates of uncertainty (e.g. confidence intervals)
☐	☒	For null hypothesis testing, the test statistic (e.g. *F*, *t*, *r*) with confidence intervals, effect sizes, degrees of freedom and *P* value noted<br>*Give P values as exact values whenever suitable.*
☒	☐	For Bayesian analysis, information on the choice of priors and Markov chain Monte Carlo settings
☒	☐	For hierarchical and complex designs, identification of the appropriate level for tests and full reporting of outcomes
☒	☐	Estimates of effect sizes (e.g. Cohen's *d*, Pearson's *r*), indicating how they were calculated

Our web collection on statistics for biologists contains articles on many of the points above.

Software and code

Policy information about availability of computer code

Data collection	MicroCal Origin, Excel, P-clamp
Data analysis	P-clamp, Microcal Origin

For manuscripts utilizing custom algorithms or software that are central to the research but not yet described in published literature, software must be made available to editors and reviewers. We strongly encourage code deposition in a community repository (e.g. GitHub). See the Nature Portfolio guidelines for submitting code & software for further information.

Data

Policy information about availability of data

All manuscripts must include a data availability statement. This statement should provide the following information, where applicable:

- Accession codes, unique identifiers, or web links for publicly available datasets
- A description of any restrictions on data availability
- For clinical datasets or third party data, please ensure that the statement adheres to our policy

The datasets generated during and/or analysed during the current study are available from the corresponding author on request and in Source Dataset

Research involving human participants, their data, or biological material

Policy information about studies with human participants or human data. See also policy information about sex, gender (identity/presentation), and sexual orientation and race, ethnicity and racism.

Reporting on sex and gender	No
Reporting on race, ethnicity, or other socially relevant groupings	No
Population characteristics	No
Recruitment	No
Ethics oversight	No

Note that full information on the approval of the study protocol must also be provided in the manuscript.

Field-specific reporting

Please select the one below that is the best fit for your research. If you are not sure, read the appropriate sections before making your selection.

[x] Life sciences [ ] Behavioural & social sciences [ ] Ecological, evolutionary & environmental sciences

For a reference copy of the document with all sections, see nature.com/documents/nr-reporting-summary-flat.pdf

Life sciences study design

All studies must disclose on these points even when the disclosure is negative.

Sample size	Power analysis: We estimate a sample size of ~7-15 experiments per group to detect at least a 15% difference between groups with=0.05 for a two-sided test to give the study >0.95 (SigmaStat, Systat Software Inc.).
Data exclusions	No data was excluded
Replication	All data were replicated
Randomization	N/A
Blinding	All in vivo and in vitro experiments were performed in a blinded fashion to remove biases. Where analyses could be performed in a "blinded" style, it was the norm in data analyses. Experiments were performed using multiple samples. Data are reported as means and standard deviations in all cases, and all outlier data values are presented. Unsuccessful experiments are documented (e.g., in electrophysiological experiments (recordings) where ionic currents and membrane potential changes are recorded, the number of cells that did not yield currents or criteria-specified viable Vrest are be stated).

Reporting for specific materials, systems and methods

We require information from authors about some types of materials, experimental systems and methods used in many studies. Here, indicate whether each material, system or method listed is relevant to your study. If you are not sure if a list item applies to your research, read the appropriate section before selecting a response.

Materials & experimental systems

n/a	Involved in the study	
[ ]	[x]	Antibodies
[ ]	[x]	Eukaryotic cell lines
[x]	[ ]	Palaeontology and archaeology
[ ]	[x]	Animals and other organisms
[x]	[ ]	Clinical data
[x]	[ ]	Dual use research of concern
[x]	[ ]	Plants

Methods

n/a	Involved in the study	
[x]	[ ]	ChIP-seq
[x]	[ ]	Flow cytometry
[x]	[ ]	MRI-based neuroimaging

Antibodies

Antibodies used	The following primary and secondary antibodies were used: Anti-GFP antibodies (Abcam), anti-mCherry antibodies (Abcam and Novous), anti-tdTomato (MyBioSouce.com), anti-Myo7a (Proteus), anti-Piezo1 (Novus), Alexa FluorTM 488 goat anti-rabbit, Alexa

	FluorTM 647 goat anti-rabbit, Alexa FluorTM 647 donkey anti-goat IgG, Alexa FluorTM 488 goat anti-mouse IgG1, Alexa FluorTM 568 goat anti-mouse IgG2a, Alexa FluorTM 568 goat anti-chicken (Invitrogen), and Phalloidin (Abcam and Sigma-Aldrich).
Validation	When available the antibody-specificity was validated using tissue from null deleted samples.

Eukaryotic cell lines

Policy information about cell lines and Sex and Gender in Research

Cell line source(s)	Neuro2A cells (N2A) ATCC (USA)
Authentication	Authenticated by Manufacturer.
Mycoplasma contamination	Cell lines tested negative for mycoplasma
Commonly misidentified lines (See ICLAC register)	N/A

Animals and other research organisms

Policy information about studies involving animals; ARRIVE guidelines recommended for reporting animal research, and Sex and Gender in Research

Laboratory animals	Mice, Piezo1-tdTomato https://www.jax.org/strain/029214, B6;129-Piezo1tm1.1Apat/J, strain #:029214; Piezo2-GFP https://www.jax.org/strain/027719, B6(SJL)-Piezo2tm1.1(cre)Apat/J, strain #:027719; Tmc1-mCherry https://www.jax.org/strain/028392, B6.Cg-Tg(Tmc1/mCherry)2Ajg/J, strain #:028392; and Tmc2-GFP https://www.jax.org/strain/028517, B6.Cg-Tg(Tmc2/AcGFP)3Ajg/J, strain #:02851; and Ai0-tdTomato https://www.jax.org/strain/007909, B6.Cg-Gt(ROSA)26Sortm9(CAG-tdTomato)Hze/J. Genotype followed primers recommended by Jackson Labs.
Wild animals	N/A
Reporting on sex	Male and Females
Field-collected samples	N/A
Ethics oversight	All animal experiments were performed under the University of Nevada Reno Institutional Animal Care and Use Committee guidelines.

Note that full information on the approval of the study protocol must also be provided in the manuscript.
